# Pig farming practices compromising biosecurity and causing poor welfare of pigs

**DOI:** 10.3389/fvets.2025.1558734

**Published:** 2025-04-17

**Authors:** Patricia Tatemoto, Fernanda Vieira, Donald M. Broom

**Affiliations:** ^1^Sinergia Animal Verein zum Schutz der Tiere, Vienna, Austria; ^2^Center for Comparative Studies in Sustainability, Health and Welfare Department of Veterinary Medicine and Animal Health, School of Veterinary Medicine, and Animal Science, FMVZ, University of São Paulo, São Paulo, Brazil; ^3^St Catharine's College and Department of Veterinary Medicine, University of Cambridge, Cambridge, United Kingdom

**Keywords:** animal welfare, extreme confinement, sustainable agriculture, swine housing, anti-microbial resistance

## Introduction

Harrison (1) reported 60 years ago on conditions in the pig production industry which did not respect the five freedoms established by the Brambell Committee a year later. Today, while in some countries pig welfare has improved, in many others the conditions are worse despite extensive research on the needs and welfare of pigs. Another example of the mismatch between science and what happens in the animal agriculture industry is the misuse of antibiotics in non-therapeutic protocols. Antimicrobial resistance poses a considerable threat to public health, with an estimated 4.95 million human deaths associated with bacterial antimicrobial resistance in 2019 (2, 3).

Responding to economic pressures, the pig industry has selected animals for increased productivity. Such modifications, combined with inadequate conditions on many farms, can have negative effects on welfare. For instance, a high number of piglets in a litter increases the competition for milk, while extreme confinement does not allow the pigs to perform their behavioral repertoire fully. These factors increase the likelihood of injurious behavior with skin lesions and other injuries as a consequence. Responses to the possibility of biting injuries in piglets or tail-biting in young pigs should be to improve the quality of the environment to meet the animals' needs, but they are often painful tooth-clipping or tail-docking. Aggression among swine females in pens occurs predominantly because of absence of opportunity for preferred behaviors such as rooting and manipulation of materials. In addition, there is competition for feed or space to establish social relations (4–6). This is also true for other farmed animals confined in artificial environments (7). Piglets reared by sows in pens show several signs of better welfare, such as displaying more play and less oral manipulative behaviors and generally have better growth rates than those reared in farrowing crates (8). It is recognized that, in the past, the motivations for the industrialization of pig farming included potential impacts on disease control and efficiency. Most available information emphasizes the benefits of intensification and supports current practices that negatively affect animal welfare, including health. Therefore, our goal was to address the other side: how such negative practices continue to be allowed despite a wealth of scientific knowledge advocating for the banning of certain methods. We aimed to highlight issues created or worsened by systems in which animals are immunosuppressed due to poor environments that offer limited opportunities for improving their welfare. While certain areas of the world, such as the European Union, have improved pig welfare, negative practices are still common in many countries, which is why a thorough investigation of harmful practices is still necessary.

## Negative impacts of sow housing and management systems on welfare

The confinement of sows in individual gestation crates simplified management and reduced space needed on farms (4, 9, 10). However, such prolonged close confinement of a cognitively complex social animal is perhaps the worst treatment of any animal by humans. There are extreme abnormalities of behavior, such as stereotypies, and a wide range of other problems for the sows, as well as impacts on the resilience of their offspring, some via epigenetic effects (8, 11–15). Confined farrowing systems prevent some sow-piglet interactions, reduce nursing duration, reduce lying behavior, and increase leg and shoulder injuries.

In addition to behavioral signs of poor welfare, keeping sows in crates or tethers impairs cardiac function, alters body conformation, reduces bone strength, and makes posture changes more difficult (16, 17). Aggression between sows in neighboring crates occurs and may have higher intensity due to the lack of opportunity to show normal social behavior (18). Lack of possibility for the sows to identify termination of agonistic interaction, results in frustration and more prolonged feelings of aggression toward neighboring sows. Lameness in immune-suppressed sows and other disease incidence can be higher in crates than in group-housing although hygiene management is a crucial factor here. Skin lesions attributed to pressure on the floor, such as decubital ulcers, are more common in crates. Sow reproductive output does not increase in crates (16).

Aggression resulting from the introduction of unfamiliar sows is a crucial challenge identified within group-housing (19). In addition to reproductive failure, aggression can also lead to lameness, feed access competition, and variable feed intake (19). Group stability minimizes this. Sows remember social events and should always be returned to the group from which they came, for example after farrowing. If sows have to be added to groups, aggression problems are reduced by a period of familiarization in adjacent pens with some degree of contact. Ample space allowance, availability of manipulable material such as bedding, and a well-designed feeding system are the critical elements in successful group-housing of post-weaned sows (19, 20). Increased space allowance during regrouping after weaning can reduce aggression and the subsequent lesions in sows (21, 22).

Since pigs were domesticated approximately ten thousand years ago (23, 24), imposed selection and management have changed them. For example, the size of the litter has increased significantly, from 4–7 offspring (25) to 10–15, reaching up to 41 piglets from a single female (26) in one exceptional case. The average number of piglets per sow per farrowing has been reported to range from 14.5 to 17 (27, 28). However, hyperprolificity in sows raises concerns regarding both sow and piglet welfare, as well as the viability of the litter due to pre-weaning losses (28). When sows are kept in a natural environment, weaning occurs between 14 and 17 weeks of age (29), whereas in commercial environments, weaning takes place as early as 3 weeks and often at 4–5 weeks. In commonly used commercial systems, female breeding pigs are inseminated for the first time at around 5–6 months of age (30). Their gestation is approximately 114 days but about 5–7 days before giving birth, the sows are moved to a farrowing crate (31). The farrowing crate makes it impossible to build a nest, which is a highly motivated behavior and they cannot express maternal behavior and interact appropriately with their offspring (32, 33). Sows often receive oxytocin to speed up the parturition (34–36). Oxytocin positively impacts situations of slow farrowing, helping to ensure piglets are born on time and reducing the risks caused by prolonged labor. However, its misuse can result in significant health risks for both sows and piglets (37). One meta-analysis demonstrated that sows that received oxytocin had an increased number of stillborn piglets compared with the sows without oxytocin administration (34).

The progeny of breeding sows are selected for rapid growth. The rapid growth demanded in pig farming requires strict feeding control for breeders. Without it, heavy animals—such as sows—living longer may develop health issues. To ensure they produce piglets with fast growth and high feed conversion rates, sows inherit these traits, which impact both them and their offspring. This means they also have an enormous appetite, but they are usually given only 50–60% of their voluntary feed intake (38). Since sows grow quickly, but feed restrictions are applied to control their weight and avoid other health problems (39), they often experience hunger during gestation. Severe feed restriction results in ongoing, unfulfilled feeding motivation (40), as pigs, in more natural conditions, spend much of their day foraging. Coping with daily hunger often leads to frustration and aggression (41). Growing pigs are slaughtered at approximately 5 to 6 months of age. However, by this time, some negative consequences of fast growth may begin to appear (42). Feed restriction protocols, often limiting intakes of sows' *ad libitum* capacity and the amount that they would choose to eat (43), are imposed to avoid obesity-related problems. This restriction causes hunger and is one of the most significant sources of stress for pregnant sows (44).

Sows may experience locomotor problems due to their fast growth and the underlying effects cause much pain. Another effect of feeding conventional concentrate feed to sows during gestation showed that they had more aggressive offspring than those from sows who received a higher fiber diet (45). Since the diet was considered equal regarding nutritional value, the authors inferred that satiety was an important variable that provided information and shaped the offspring during its development. Feed restriction in the prenatal environment may shape the offspring to be more competitive, resulting in more aggressiveness (45). This may be an evolutionary attempt to better prepare piglets for a postnatal environment where there are disputes over resources and in which aggressiveness would be a phenotype that could increase survival rates. Such an adaptation has unintended negative effects in intensive farming, especially in crowded high-density pens without positive stimuli.

## Piglet welfare, including health

The environment in which an animal is maintained during gestation may result in changes in several offspring qualities (46–50). Challenges in the prenatal and neonatal periods can modulate factors such as emotional reactivity, responsiveness to stressors, and cognition (49, 51). Thus the prenatal environment of the piglets has been proven to affect their welfare, including aspects of their health, as well as impacting the organization of the central nervous system (11–15). Poor and barren conditions, such as gestation crates or environments with low positive stimuli that do not fulfill sows' needs, can negatively impact piglet development. The poor conditions in which boars are kept during sperm cell development can also affect piglet development (52).

Research has documented the consequences of painful practices carried out by the swine industry during the initial days of a piglet's life (53–55). It is clear that ear notching, teeth clipping, hot cautery tail docking, and tearing during castration result in increased pain (53). Cutting piglets' teeth is a practice adopted by the industry to avoid problems generally caused by the stressful conditions in which these animals are raised (56). Overcrowding in sheds and environments lacking positive stimuli can encourage aggressive behavior in piglets and growing pigs (5, 6, 57). There do not appear to be systematic records of piglet facial or sow teat lesions for semi-natural environments (56), suggesting that these problems are not important in such environments but are exacerbated by the stressful conditions to which animals in widely-used conditions are exposed. A reason used to justify the teeth-clipping mutilation is that piglets' teeth can cause injuries to sows' mammary areas (56, 58). The farrowing system and flooring type are known to be risk factors for piglet facial and sow mammary lesions (56). In addition, the number of piglets per sow has been manipulated to increase over the years, generating increased competition among piglets. However, some studies have shown that good management practices can make routine teeth cutting unnecessary. For instance, an enriched environment in early life can reduce piglet facial and sow teat lesions (56, 59). Although banned in the EU since 2008 (60), these practices remain permitted in many parts of the globe despite all scientific evidence supporting a ban.

Other widely used practices that cause pain in newborn piglets are ear-notching for identification (61) and surgical castration without pain control (53). These practices are contrary to the Federal Constitutions, legislative frameworks, and Veterinarian Federal Council standards of several countries, including Brazil (62, 63). Future protocols must not violate legal instruments. There are ways to avoid pain and the experience of intense pain should not be accepted (64).

## Biosecurity risks and the misuse of antimicrobials

As evidenced by Schuck-Paim and Alonso (64), although large animal production facilities can rely on various biosecurity protocols and sanitary standards to prevent and control the potential transmission of infectious diseases and resistant bacteria, for example, flaws in biosecurity practices are widespread, even in countries where compliance is expected to be higher, such as Sweden, Canada, the United States and Australia (65, 66). For instance, nearly 1.5 billion pigs are slaughtered for food worldwide every year, or about 4 million per day. Pre-slaughter mortality rates in the industry are about 5%−10% during the suckling phase, 3% following weaning and an extra 3% during growth (67). This translates into about half a million carcasses of dead pigs per day that must be disposed of. Not every farm will have the proper means of ensuring that dead carcasses (often of sick animals) are disposed of following the proper biosecurity standards. The situation worsens during infectious disease outbreaks when the number of animals that must be culled often exceeds the capacity of proper disposal and recycling facilities. In these cases, it is not uncommon for hundreds of animals to be hauled to landfills.

Diarrhea in weaned piglets may occur because of: weaning too early, for example at 3–4 weeks (29), or abrupt diet change from sow milk to solid feed, perhaps combined with immunosuppression caused by chronic stress (68–70). As a result, antimicrobials are misused in pig farming and other livestock contexts. More than 70% of antibiotics sold worldwide are used for animals raised on farms (71). Brazil, for example, is the second largest consumer of antibiotics in the world, surpassed only by China (71). While the European Union has already banned the indiscriminate use of these drugs in livestock farming, there are no prohibitions or controls on the use of antimicrobials in swine for prophylactic and metaphylactic purposes in Brazil (72). This is evidenced by the fact that Brazilian industry giants such as BRF, JBS, and Aurora Alimentos continue to permit the use of antibiotics on healthy animals on a large scale (73).

Oral medication in suckling and post-weaning periods is the most common application of antibiotic administration in pig production (74). The livestock industry's use of antimicrobials, including antibiotics, as growth promoters threatens public health as it increases the risk of Antimicrobial Resistance (AMR) (3, 75, 76). AMR occurs when bacteria, viruses, fungi, and parasites are no longer controlled by antimicrobial agents. As a result, infections become difficult or impossible to treat, increasing the risk of disease spread, severe illness, and death. AMR poses a considerable threat to public health, with an estimated 5 million human deaths associated with bacterial antimicrobial resistance per year (2, 3).

Antimicrobials, including antibiotics, are misused in human medicine and on billions of animals in the livestock industry to prevent infections resulting from precarious sanitary conditions, high housing densities, and the fragile health of genetically selected animals (64). The EIP-AGRI Network for Agriculture and Innovation, funded by the European Commission, identified the main interrelated areas of intervention for reducing antibiotic use, the first of which is the general enhancement of animal welfare (77). Better welfare leads to better immune system function and less disease.

Genetic selection aimed at productivity also worsens the situation because the energy that animals would allocate to immune defense is directed toward growth and reproduction (78, 79). The animals' increased vulnerability to infections represents not only a risk of the emergence of highly pathogenic viral or bacterial strains but also a significant risk to food safety, increasing the probability of diseases caused by enteric pathogens (64).

## Solutions for the pig industry

Pigs can be reared so that their welfare is good. The methods involve ensuring that the needs of the pigs are met and that harms to pigs are avoided. Welfare is an important part of the sustainability of production systems and good welfare is demanded more and more by consumers (17). Changes in the pig industry to become more sustainable (80) will increase the chances of the survival of the industry (81).

## Conclusion

The urgent need to move toward a more acceptable business model in the livestock sector is uncontroversial. Many current widespread practices not only fail to comply with countries' legal frameworks but are also unethical based on the scientific knowledge available, in part because they also compromise public health. Our paper challenges the prevailing status quo of large-scale intensive pig production farming, but it is time that animal welfare and agriculture policies should be made in line with the science that shows how damaging intensive confinement systems are to animals, human health, and the environment. The pig industry can change to become more sustainable and pig welfare is an important component of a sustainable future industry.
